# The Novel Omega-6 Fatty Acid Docosapentaenoic Acid Positively Modulates Brain Innate Immune Response for Resolving Neuroinflammation at Early and Late Stages of Humanized APOE-Based Alzheimer's Disease Models

**DOI:** 10.3389/fimmu.2020.558036

**Published:** 2020-10-16

**Authors:** Qiu-Lan Ma, Cansheng Zhu, Marco Morselli, Trent Su, Matteo Pelligrini, Zhengqi Lu, Mychica Jones, Paul Denver, Daniel Castro, Xuelin Gu, Frances Relampagos, Kaitlin Caoili, Bruce Teter, Sally A. Frautschy, Gregory M. Cole

**Affiliations:** ^1^Department of Neurology, University of California, Los Angeles, Los Angeles, CA, United States; ^2^Geriatric Research and Clinical Center, Greater Los Angeles Veterans Affairs Healthcare System, West Los Angeles VA Medical Center, Los Angeles, CA, United States; ^3^Department of Neurology, The Third Affiliated Hospital, Sun Yat-sen University, Guangzhou, China; ^4^Department of Molecular, Cell and Developmental Biology, University of California, Los Angeles, Los Angeles, CA, United States; ^5^Institute for Genomics and Proteomics, University of California, Los Angeles, Los Angeles, CA, United States; ^6^Institute for Quantitative and Computational Biology, University of California, Los Angeles, Los Angeles, CA, United States; ^7^Department of Biological Chemistry, University of California, Los Angeles, Los Angeles, CA, United States; ^8^Molecular Biology Institute, University of California, Los Angeles, Los Angeles, CA, United States; ^9^Department of Medicine, University of California, Los Angeles, Los Angeles, CA, United States

**Keywords:** Alzheimer's disease, neuroinflammation, fatty acid, linoleic acid, docosapentaenoic acid, APOE, EFAD

## Abstract

Neuroinflammation plays a crucial role in the development and progression of Alzheimer's disease (AD), in which activated microglia are found to be associated with neurodegeneration. However, there is limited evidence showing how neuroinflammation and activated microglia are directly linked to neurodegeneration *in vivo*. Besides, there are currently no effective anti-inflammatory drugs for AD. In this study, we report on an effective anti-inflammatory lipid, linoleic acid (LA) metabolite docosapentaenoic acid (DPAn-6) treatment of aged humanized EFAD mice with advanced AD pathology. We also report the associations of neuroinflammatory and/or activated microglial markers with neurodegeneration *in vivo*. First, we found that dietary LA reduced proinflammatory cytokines of IL1-β, IL-6, as well as mRNA expression of COX2 toward resolving neuroinflammation with an increase of IL-10 in adult AD models E3FAD and E4FAD mice. Brain fatty acid assays showed a five to six-fold increase in DPAn-6 by dietary LA, especially more in E4FAD mice, when compared to standard diet. Thus, we tested DPAn-6 in aged E4FAD mice. After DPAn-6 was administered to the E4FAD mice by oral gavage for three weeks, we found that DPAn-6 reduced microgliosis and mRNA expressions of inflammatory, microglial, and caspase markers. Further, DPAn-6 increased mRNA expressions of ADCYAP1, VGF, and neuronal pentraxin 2 in parallel, all of which were inversely correlated with inflammatory and microglial markers. Finally, both LA and DPAn-6 directly reduced mRNA expression of COX2 in amyloid-beta42 oligomer-challenged BV2 microglial cells. Together, these data indicated that DPAn-6 modulated neuroinflammatory responses toward resolution and improvement of neurodegeneration in the late stages of AD models.

## Introduction

Neuroinflammation, derived from innate immune responses, is commonly characterized by the release of inflammatory cytokines from activated microglia. It substantially contributes to the pathogenesis and progression of Alzheimer's disease (AD) (1). The important role of neuroinflammation in AD is underscored by findings in recent years from human genome-wide association studies, whole-genome sequencing, and gene expression network analysis, which have uncovered common and rare genetic variants that are associated with AD. These genes are involved in innate immunity, implicating microglial genes in the development and progression of AD such as TREM2, CD33, TYROBP, and other disease-associated microglia genes (2–6). In addition, environmental factors also influence inflammatory responses that could contribute to neuroinflammation and pathologies of AD such as systemic infection, obesity, type 2 diabetes mellitus, coronary artery disease, trauma, and dietary habits (7). In AD, a sustained chronic neuroinflammatory response can not only be toxic to neurons but also decrease the capability of microglia to phagocytize or degrade Aβ, causing further exacerbation of AD pathologies. Thus, inhibition of inflammation by either effective anti-inflammatory drugs or limiting environmental risk factors could be an important preventive and therapeutic strategies for AD.

Epidemiological and animal studies have shown convincing evidence that nonsteroidal anti-inflammatory drug (NSAID) use is associated with risk reduction for AD in humans and AD pathology in animal models (8, 9). In an early longitudinal study of aging which included 1,686 participants, a 50% reduction of AD was associated with consistent NSAID consumption (10). In subsequent studies, more than 15 epidemiological studies have reported a consistent finding that the longer the NSAIDs were used prior to clinical diagnosis, the greater the sparing effect (8). NSAIDs are typically competitive inhibitors of cyclooxygenase (COX), the enzyme that mediates the bioconversion of arachidonic acid to inflammatory prostaglandins (PGs) (11). However, clinical trials with NSAIDs have generally not shown positive results for treating or preventing AD (12), suggesting the complexity of the inflammatory process in AD and a need to explore other effective anti-inflammatory or immunomodulatory agents.

Extensive studies have suggested that omega-6 arachidonic acid is primarily pro-inflammatory. However, long-chain omega-3 (n-3) polyunsaturated fatty acids (PUFAs), namely docosahexaenoic acid (DHA) and eicosapentaenoic acid (EPA), as well as their enzymatically oxygenated mediators such as protectins, resolvins, and maresins have anti-inflammatory effects in AD (13–15). In many studies, the ratio of n-3 to n-6 has been often used as an index to reflect the relative abundance of n-3 PUFAs. The role of n-6 PUFAs has not been as well studied. It is typically considered pro-inflammatory although some n-6 PUFAs exhibit effects similar to n-3 PUFA in some studies (16). Therefore, we explored the role of dietary linoleic acid (LA) in neuroinflammation and investigated its possible anti-inflammatory mechanisms in AD models. Because the most altered CNS n-6 fatty acid metabolite on LA diet in EFAD mice was docosapentaenoic acid (DPAn-6) which can serve as a substrate for anti-inflammatory resolving lipid mediators, we tested the impact of daily direct gavage administration of DPAn-6 on neuroinflammation in late stages of a severe AD pathology model E4FAD (APOE4-TR^+/+^/5xFAD^+/−^) mice for three weeks, which carries human APOE4 targeted replacement (APOE4-TR^+/+^). We found that DPAn-6, a long-chain metabolite of LA, has anti-neuroinflammatory and neuroprotective effects in late stages of E4FAD mice. Our results provide the first evidence of DPAn-6 control of neuroinflammation in AD models.

## Materials and Methods

Ethics Statement—All animal experiments were conducted with the approval of the Animal Research Committee, University of California, Los Angeles and carried out with strict adherence to the current guidelines set out in the NIH Guide for the Care and Use of Laboratory Animals at the Association for Assessment and Accreditation of Laboratory Animal Care.

### High Omega-6 (n-6) Linoleic Acid Diet in Adult E3FAD and E4FAD Mice

Since APOE4 is a strong genetic risk factor for AD (17), we used E3FAD and E4FAD mice for this study. These EFAD mice were originally obtained from Dr. Mary Jo Ladu (University Illinois, Chicago). They were made by crossing human APOE-target replace (APOE-TR^+/+^) mice on a C57/BL6 background with 5xFAD mice^+/−^(18). The 5xFAD transgenic mice developed amyloid plaques at the age of 2-month old and markedly over-produce human Aβ42 (19), the most abundant peptide found in AD amyloid plaque pathology. In EFAD mice, Aβ deposition was delayed by about 4 months compared to 5xFAD mice (20). E4FAD mice, analogous to human APOE4-carriers, have more accelerated AD pathology than E3FAD mice (20). In addition, APOE4-TR mice have impaired lipid metabolism (21). In this study, we tested whether high n-6 linoleic acid (LA) diet has a different impact on APOE isoforms with E3FAD and E4FAD mice. The EFAD mice of both sexes were raised on a standard diet (ST, Purina 5015 breeder chow; Purina Mills, St. Louis, MO) until 3–4-months. then, the mice were split into two groups. Two groups (high LA diet, *n* = 8/per group, 5 females, 3 males for both E3FAD and E4FAD mice, respectively) were switched to LA-enriched diet using safflower oil that delivered 4.86% of LA or 6% of total fat in mouse food diet (weight/weight) (Harlan Teklad, Madison, WI) for 4 months. The other two groups (ST diet, *n* = 9/per group, 5 females, 4 males for both E3FAD and E4FAD mice, respectively) remained on ST diet (5015 breeder chow) as controls.

### Administration of n-6 Docosapentaenoic Acid (DPAn-6) by Oral Gavage to Aged E4FAD Mice

In the high LA diet experiment, we found that a high LA diet increased its metabolite docosapentaenoic acid (DPAn-6) in the brain, which was increased more in E4FAD mice than in E3FAD mice. Thus, we further tested whether DPAn-6 *per se* had a treatment effect in aged E4FAD mice. During this experiment, DPAn-6 was administrated daily by oral gavage to 12 to 14- months old E4FAD mice (*n* = 5, 3 females, 2 males) at 700 mg/Kg body weight for three weeks. They were then compared to age-matched control vehicle-treated mice (*n* = 7, 4 females, 3 males) that received oral gavage with vehicle containing 0.1% of antioxidants of alpha-tocopherol and 0.1% of palmitoyl ascorbic acid. These antioxidants provided to all groups were used to prevent the *ex vivo* lipid peroxidation of the DPAn-6 (ethyl ester) preparation. DPAn-6 was purchased from Nu-Chek Prep Inc.

### Animal Brain Tissue Collection

At the end of the experiment, the mice were sacrificed under deep anesthesia and perfused by TBS buffer with protease and phosphatase inhibitors. Different brain regions were dissected from one hemisphere for biochemistry. The other brain hemisphere was fixed in 4% paraformaldehyde and sectioned for immunohistochemistry as previously described (22).

### Brain Fatty Acid Analysis

Brain fatty acid analysis was conducted on the frontal cortex using the Folch method and gas chromatography with flame ionization detection as reported previously (23, 24).

### Brain Proinflammatory Cytokines Assay

An MSD V-Plex cytokine panel assay was performed on brain hippocampal tissues using a 10-multi-spot 96 well plate according to the manufacturer's instructions. The assay is a multiplex sandwich immunoassay. Among the 10 capture antibodies, only interleukin 1 beta (IL-1β), interleukin 5, interleukin 6 (IL-6), interleukin 8, interleukin 10 (IL-10), and tumor necrosis alpha (TNFα) were reliably detected in hippocampus. Bovine serum albumin coating was used to reduce non-specific binding. Cytokine levels were measured using duplicate determinations for standards and samples and then analyzed by an MSD SECTOR Imager.

### Immunohistochemical Staining, Light Microscopy, and Confocal Microscopy

Frozen formalin-fixed mouse brains were sectioned for coronal sections at a thickness of 12 μm, mounted on pre-cleaned and pre-coated Superfrost Plus slides (Fisher Scientific), dried overnight, and slides were stored in sealed boxes in a −20°C freezer for two more days to allow cold drying before use. For 3,3'-diaminobenzidine (DAB) staining, slides with sections were stained for Ionized calcium-binding adaptor molecule 1 (Iba1, Wako Chemicals USA, Inc., Richmond, VA) and evaluated by light microscopy. Stored slides with cryosections of mouse brain were removed from −20°C freezer, warmed to room temperature for 20 min, then steamed for 15 min in Unmasking Antigen Solution. Sections were quenched in 0.6% hydrogen peroxide with methanol for 30 min at room temperature and washed 3 times with TBS. After treating with 0.3% Triton X-100 in TBS for 10 min, sections were incubated in a blocking solution with 5% normal goat serum and 3% BSA in TBS at 37°C for one hour. Then the primary antibody (1:200 dilution) was added to sections, and they were incubated at 4°C overnight. Vector biotinylated goat anti-rabbit (BA-1000) antibody (1:1,200) was used with 1.5% normal goat serum and 3% bovine serum albumin (BSA) in TBS. Then sections were incubated in secondary antibody followed by VECTASTAIN ABC Elite Standard Kit (PK-6100, Vector Labs) at 37°C for one hour. Sections were developed using a Peroxidase/Diaminobezidiene Metal Enhance Substrate kit (Cat. 34065; Pierce, Rockford, IL.). The images were taken every 10 sections through the hippocampus and analyzed with ImageJ. First, the images of layers 4–6 of the temporal cortex are acquired in TIFF format at 10x magnification on a Leica digital MC170HD camera with 5M pixels, attached to a Nikon Eclipse E800 microscope. Then the macros for Image J are run. The first macro calibrates the image to micrometers and optical densities (OD) from 0 (white) to 2.6 (black), runs “RGB stack,” enhances contrast at saturation 0.2%, then smooths stacks and set threshold range for 1.65 to 2.55 OD. The second macro analyzes particles in the green channel slices, which best picks up the brown microglia images. Variables measured are “count” (# of particles in selected region), average particle size (μm^2^), percentage area, total area (all particle areas in field). Then the “summary clipboard” of average values are pasted into excel. The region of interest area (ROI) is calculated by (100^*^total area/%area). Cell density is expressed as cells/1000 μm^2^ and calculated by (Count/RO1).

For immunofluorescence staining, after additional slides with sections were removed from the freezer and allowed to equilibrate to room temperature, they were immersed in distilled water for 5 min followed by Vector antigen unmasking solution (cat# H-3300) and steamed for 15 min. After washing in Tris buffer solution (TBS); 0.3% triton X-100 in TBS for 10 min, sections were incubated at 37°C for one hour in blocking solution containing 5% normal goat serum and 3% BSA in TBS. Sections are incubated with the first primary antibody anti-caspase cleaved actin (fragment of actin or “Fractin”) at a dilution to 1: 100 at 4°C overnight, then followed by incubation with the secondary antibody tagged with a fluorescent dye (Red, Alexa Flour plus 594) at a dilution to 1:1200 at 37°C for one hour. Fractin antibody was developed in our lab to detect end-specific cleavage by caspase-3 or caspase-6 (25). Finally, sections were counterstained with DAPI dye to stain nuclei by VECTASHIELD Antifade Mounting Medium with DAPI. To minimize quenching of the signal, sections were stored in the dark during staining, and examined at The UCLA Confocal Microscopy Core using LEICA Confocal Microscope (SP2 1P-FCS) and then analyzed with NIH-Image public domain software ImageJ. The fluorescence macros were the same as above analyzing Iba1 except using “setAutoThreshold” (“Default dark”) and setThreshold (166, 255, “raw”).

### RNA Purification and Library Preparation

Total RNA was isolated from mouse brain cortex tissue or harvested cultured cells using Ambion RNAqueous® phenol-free total RNA isolation kit with DNase treatment according to the manufacturer's instructions (Ambion Inc., Austin, Texas, USA). DNA libraries were prepared using KAPA mRNA (Roche) according to the manufacturer's instructions and sequenced on Hiseq3000 at the UCLA Technology Center for Genomics & Bioinformatics.

### RNA-Sequencing (RNA-Seq) Processing and Analysis

RNA-Seq reads (generated using STAR quantmode) were aligned using STAR 2.5 (26) to the mouse reference genome ENSEMBL (version GRCm38.88) and read counts per gene “GeneCounts” were generated in a gtf file. Differential gene expression analysis was performed using the *voom* method that is designed to accommodate the mean-variance relationship of the transformed data using precision weights (Part of limma-voom method). Limma-voom is a widely used method for the RNA-Seq differential gene expression analysis (27). We defined a select subset of treatment-affected genes based on a two-tailed *t*-test (*p* < 0.05) (28) and whether these genes were decreased or increased by DPAn-6.

### cDNA Synthesis and Quantitative Real-Time Reverse Transcription PCR (RT-PCR)

RETROscript™ first strand synthesis kit was used for reverse transcription of cDNA synthesis (Ambion Inc., Austin, Texas, USA). Gene mRNA levels of cyclooxygenase-2 (COX-2) and hypoxanthine guanine phosphoribosyl transferase (HPRT) were measured by RT-qPCR (TaqMan technology) in 384 well plates using an ABI 7900HT real-time PCR machine. Pre-designed TaqMan Rodent primers and probes were used for the assay (Thermo Fisher Scientific). The HPRT was used as an internal control to normalize the relative expression of COX-2.

### Microglial BV2 Cell Culture and Treatments

Immortalized BV2 murine microglial cells were gifted from Dr. Monica J. Carson (University of California, Riverside). Cells were cultured in 6 well plates in Dulbecco's modified Eagle's medium (DMEM, Gibco-BRL, Grand Island, NY, USA) supplemented with 10% heat-inactivated fetal bovine serum (Gibco), 100 U/mL penicillin, 100 μg/mL streptomycin and 2 mM L-glutamine in a 5% CO_2_-humidified air environment incubator at 37°C. BV2 cells were pretreated with or without 50 μM of n-6 docosapentaenoic acid or linoleic acid in DMEM culture media with 2% serum for 24 h. The cells were then changed to a serum-free media of DMEM with or without 0.25 μM of Aβ42 oligomers for an additional one or four hours. Tocopherol acetate (10 μg/mL) was used as an antioxidant to prevent *ex vivo* lipid peroxidation for all groups including controls. At the end of the experiments, the BV2 cells were collected using a lysis buffer (provided in the Ambion RNAqueous® phenol-free total RNA isolation kit) for RNA extraction and analysis. COX-2 and HPRT mRNA expressions were measured by RT-qPCR as described above.

### Statistical Analysis

Experimental data were presented as group means ± SEM. RNA-Seq expression unit was normalized as log counts per million (log CPM). RNA-Seq data distribution was assumed to be normal but this was not formally tested (29). Law et al. proposed the *voom* transformation to transform the count distribution to a distribution close to the normal distribution in RNA-Seq data analysis and demonstrated that using *limma* with the *voom*-transformed count data performed comparable to count-based RNA-Seq analysis methods, such as edgeR, DESeq, baySeq, and DSS (27, 30). Recent evaluation of the limma-voom method ranks it among the top performing RNA-Seq differential expression analysis tool, and Limma+voom is one of the most balanced software programs considering the precision, accuracy, and sensitivity that it delivers (31). In addition, the published methods article using limma-voom tutorial specifies that it does not require testing of transformed read counts for normality of the distribution (29). The *voom* transformation is a sample-specific transformation, defined as log-CPM (30). Two tailed Student *t*-test (unpaired) was used to compare two groups. One-way ANOVA was used to compare more than two groups using GraphPad Prism software. Experimental animals were coded. Experiments conduct, data collection, and analysis were performed with the experimenter blind to the assigned groups.

## Results

### Impact of Omega-6 Linoleic Acid-Enriched Diet on Brain Polyunsaturated Fatty Acids in EFAD Mice

There are two essential classes of polyunsaturated fatty acid (PUFAs) that humans cannot synthesize. They must be obtained from food. One series is omega-3 (n-3) that begins with alpha linolenic acid (ALA, 18:3) which is used for the synthesis of long-chain n-3 PUFAs such as DHA and EPA, which can be metabolized to anti-inflammatory eicosanoids to reduce the risks for AD, cardiovascular diseases and other chronic inflammatory diseases (32–34). The other essential PUFA is n-6 linoleic acid (LA, 18:2) which is used for the synthesis of long-chain n-6 PUFAs. Physically both n-3 and n-6 PUFAs are important constituents of cell membranes influencing protein mobility while their enzymatically oxidized metabolites influence cellular function via membrane-bound receptors. The n-6 PUFAs have often been assumed to have a proinflammatory function but this has not been as extensively studied in CNS as n-3 PUFAs. To begin to understand whether a high LA diet had an impact on neuroinflammation locally in the brain, we evaluated whether high LA diet altered brain fatty acid composition. The fatty acids are expressed as % of total fatty acids. Table 1 shows that a high n-6 diet did not alter brain LA levels. However, it dramatically increased its metabolite, n-6 docosapentaenoic acid (DPAn-6, 343% in E3FAD, *p* < 0.0001; 574% in E4FAD, *p* < 0.0001) compared to standard diet (ST) diet in C57BL/6 mice. In contrast, the high n-6 diet only slightly increased arachidonic acid levels (ARA, 9.6% in E3FAD, *p* < 0.0001; 8.4% in E4FAD, *p* < 0.001) and docosatetraenoic acid (DTA, 20.9% in E3FAD, *p* < 0.0001; 25% in E4FAD, *p* < 0.0001). In addition, high LA diet also slightly reduced n-3 docosahexaenoic acid (DHA, 7.6% in E3FAD mice, *p* < 0.001; 12.8% in E4FAD mice, *p* < 0.0001) and dihomo-gamma-linoleic acid (DGLA, 25.1% in E3FAD, *p* < 0.0001; 33.3% in E4FAD, *p* < 0.0001). These data clearly indicate that a high LA diet affects the brain fatty acid content more in E4FAD mice than in E3FAD mice, possibly due to increased n-6 turnover by higher amyloid beta and inflammation-driven cytosolic phospholipases. These data also show that the brain level of DPAn-6 was significantly more increased than other PUFAs due to high LA intake in these EFAD mice while DHA showed a modest loss. As a result, we decided to study the impact of DPAn-6 on neuroinflammation in aged E4FAD mice. DPAn-6 (22:5n-6) has a similar structure to the n-3 DHA (22:6n-3), which also has 22 carbons and ≥ 3 double bonds; the main difference from DHA is the single carbon-carbon double bond at the Δ19 position.

**Table 1:** Polyunsaturated fatty acids (PUFAs) content in frontal cortex of EFAD mice.

		**n-6 PUFAs**			**n-3 PUFAs**		
**Diet**	***N***	**LA 18:2n-6^(1)^**	**DGLA 20:3n-6^(2)^**	**ARA 20:4n-6^(3)^**	**DTA 22:4n-6^(4)^**	**DPA 22:5n-6^(5)^**	**DHA 22:6n-3^(6)^**
ST diet in C57BL/J mice	9	0.57 ± 0.016	0.344 ± 0.006	9.8 ± 0.084	2.44 ± 0.61	0.23 ± 0.009	17.24 ± 0.179
LA diet in E3FAD mice	8	0.54 ± 0.021	0.275 ± 0.014^****^	10.74 ± 0.168^****^	2.95 ± 0.055^****^	1.02 ± 0.120^****^	16.02 ± 0.291^***^
LA diet in E4FAD mice	8	0.61 ± 0.020	0.258 ± 0.011^****^	10.62 ± 0.128^***^	3.05 ± 0.028^****^	1.55 ± 0.087^****^	15.278 ± 0.155^****^

*Values are expressed as mean % of total frontal cortex fatty acid ± SEM. The comparison is high LA diet vs. standard (ST) diet*.

*** *P < 0.001*,

**** *P < 0.0001*.

(1) *linoleic acid (LA)*,

(2) *dihomo ⋎-linolenic acid (DGLA)*,

(3) *arachidonic acid (ARA)*,

(4) *docosatetraenoic acid (DTA)*,

(5) *docosapentaenoic acid (DPA)*,

(6) *docosahexaenoic acid (DHA)*.

### Modulation of Brain Pro-inflammatory Cytokines and Cytokine Receptors With Omega-6 Fatty Acids in EFAD Mice

**Figure 1:**
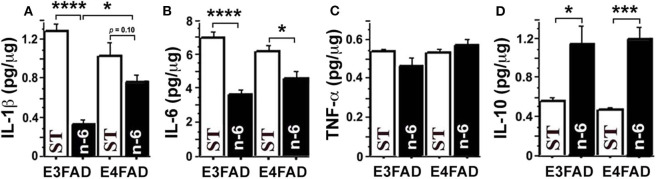
The high n-6 linoleic acid diet reduced brain hippocampal tissue pro-inflammatory cytokines in EFAD mice. **(A)** High linoleic acid (n-6 or LA) diet reduced brain pro-inflammatory cytokine IL-1β in E3FAD (*p* < 0.0001) and a trend in E4FAD mice (*p* = 0.1) compared to the EFAD mice on standard (ST) diet. **(B)** High n-6 diet reduced pro-inflammatory cytokine IL-6 in E3FAD (*p* < 0.0001) and E4FAD mice *(p* < 0.5). **(C)** High n-6 diet did not change pro-inflammatory cytokine TNF-α. **(D)** High n-6 diet increased the inflammation-resolving cytokine IL-10 in both E3FAD (*p* < 0.05) and E4FAD mice (*p* < 0.001). Data represent the mean ± SEM. Significance was determined by one-way ANOVA, *p < 0.05, ***p < 0.001, and ****p < 0.0001.

To test whether a high linoleic acid (LA) diet had an impact on neuroinflammation in EFAD mice, we measured proinflammatory cytokines from brain hippocampal tissue with an MSD V-Plex multiplex ELISA kit panel. Surprisingly, we found that a high LA diet, which was initially predicted to increase pro-inflammatory arachidonic acid products and inflammation, actually significantly reduced several pro-inflammatory cytokines and increased anti-inflammatory cytokines. Figure 1 shows that high n-6 diet reduced brain pro-inflammatory cytokines IL-1β (*p* < 0.0001 in E3FAD, a trend *p* = 0.1 in E4FAD mice, Figure 1A) and IL-6 (*p* < 0.0001 in E3FAD, *p* < 0.05 in E4FAD mice, Figure 1B) compared to the standard (ST) diet in EFAD mice. TNF-α had no significant changes (*p* > 0.05, Figure 1C). Interestingly, the inflammation-resolving cytokine IL-10 was increased by high LA diet in both E3FAD (*p* < 0.05) and E4FAD mice (*p* < 0.001, Figure 1D). This data suggested that a high LA diet promoted resolution of neuroinflammation in EFAD mice.

Because the high LA diet dramatically increased brain levels of DPAn-6 but did not alter the levels of LA in EFAD mice, we speculated that dietary LA's impact on neuroinflammation might be through its metabolite DPAn-6.

**Figure 2:**
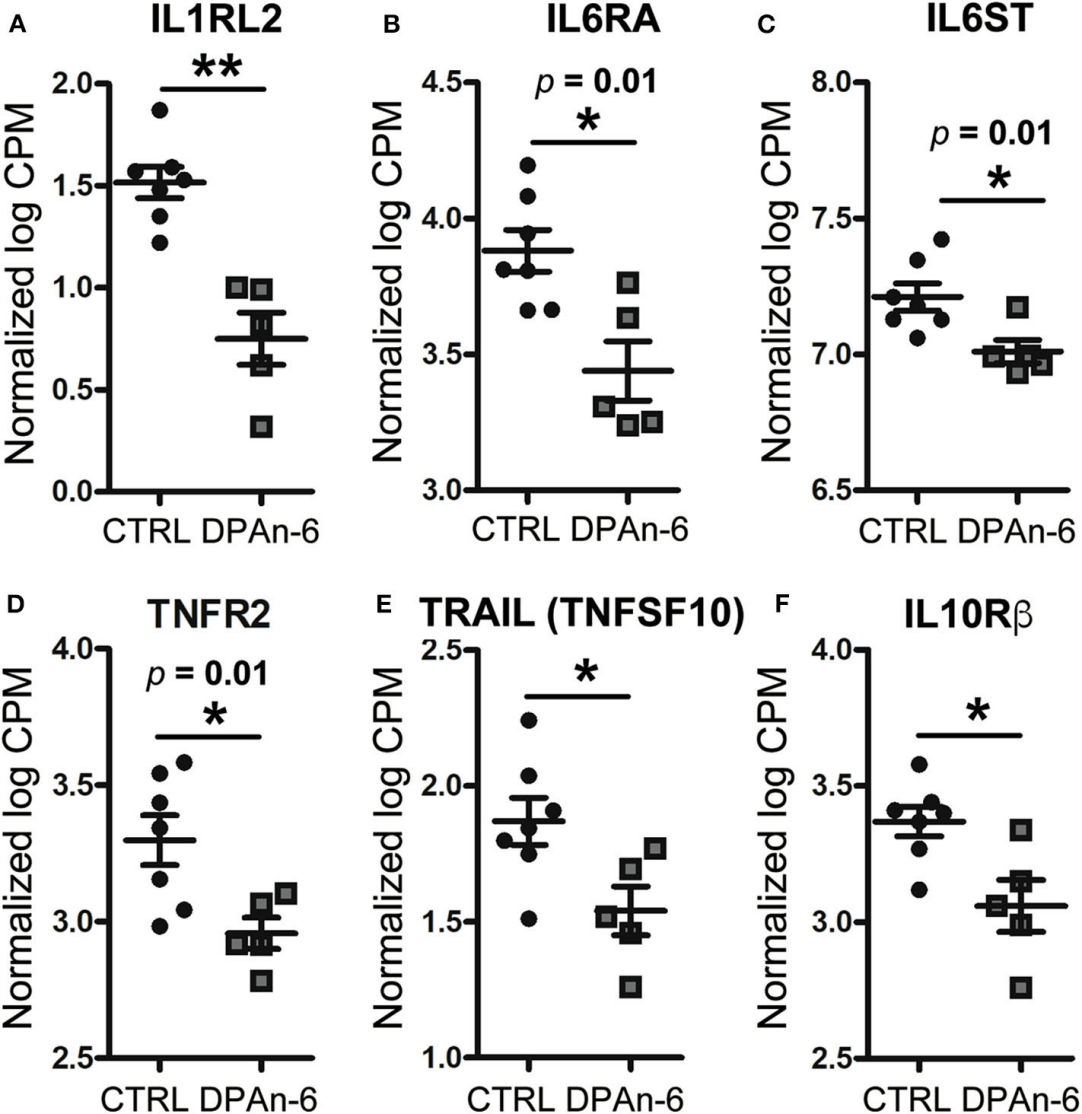
DPAn-6 reduced mRNA expression of proinflammatory cytokine and cytokine receptors in the brain of aged E4FAD mice. In RNA-Seq data, **(A)** DPAn-6 reduced interleukin-1 receptor-like 2 (ILRL2, *p* < 0.01). **(B)** DPAn-6 reduced interleukin 6 receptor alpha (IL6RA, *p* = 0.01). **(C)** DPAn-6 reduced IL6 signal transducer (IL6ST, *p* = 0.01). **(D)** DPAn-6 reduced tumor necrosis factor receptor 2 (TNFR2, *p* = 0.01). **(E)** DPAn-6 reduced tumor necrosis factor-related apoptosis-inducing ligand (TRAIL, also known as tumor necrosis factor superfamily, member 10 (TNFSF10), *p* < 0.05)). **(F)** DPAn-6 decreased IL-10 receptor beta (IL-10Rß, *p* < 0.05). The RNA-Seq data were normalized by Count Per Million (CPM) and presented as Log2 expression. Data represent the mean ± SEM. Significance was determined using an unpaired, two-tailed Student's *t*-test, *p < 0.05, and **p < 0.01.

To understand the role of DPAn-6 in neuroinflammation, 12 to 14-month-old E4FAD mice were treated by oral gavage DPAn-6 for three weeks. Brain total RNA was extracted from the cortex for RNA sequencing (RNA-Seq) analysis. Our results show that DPAn-6 reduced mRNA expression of interleukin-1 receptor-like 2 (IL1RL2, *p* < 0.01, Figure 2A), interleukin 6 receptor alpha (IL6RA, p = 0.01, Figure 2B), IL6 signal transducer (IL6ST, *p* = 0.01, Figure 2C), tumor necrosis factor receptor 2 (TNFR2, *p* = 0.01, Figure 2D) and tumor necrosis factor-related apoptosis-inducing ligand (TRAIL, also known as tumor necrosis factor superfamily, member 10 (TNFSF10), *p* < 0.05, Figure 2E). These inflammatory markers are significantly upregulated in the AD brain and implicated in the pathogenesis of many diseases. IL1RL2 is strongly expressed in the vicinity of Aβ plaques and neurofibrillary tangles in AD (35). IL-6 receptor (IL6RA) is upregulated in AD frontal and occipital cortex (36). IL6ST is a constituent of a cell-surface type I cytokine receptor complex required in the IL-6 signaling cascade that contributes to neuroinflammation in AD (36). High levels of TNFR1 and TNFR2 in CSF are associated with the conversion of mild cognitive impairment (MCI) to dementia (37). In addition, TNFR1 is required for Aβ-induced neuron death in mouse AD model brains (38). Interestingly, DPAn-6 also decreased IL-10 receptor beta (IL-10Rβ, *p* < 0.05, Figure 2F). IL-10 is a key component of anti-inflammatory signaling that inhibits proinflammatory responses to resolve inflammation in most early disease conditions (39). However, IL-10 is upregulated in AD brain and serum (40–42), and IL-10 knockout preserves synaptic integrity and mitigates cognitive disturbance in APP/PS1 mice (43). IL-10Rβ mRNA expression is also upregulated in AD patient brain detected by cDNA microarrays (44). Our data here suggest that DPAn-6 might modulate the neuroinflammatory response toward resolution and homeostasis from severe disease conditions. Consistently, DPAn-6 inhibited LPS-stimulated elevation of IL-1β and TNFα in human peripheral blood mononuclear cells and acute rat paw edema *in vivo* (45), providing similar direct evidence for the DPAn-6 modulation of inflammation toward resolution.

### Attenuation of Microgliosis With DPAn-6 in Aged E4FAD Mice

**Figure 3:**
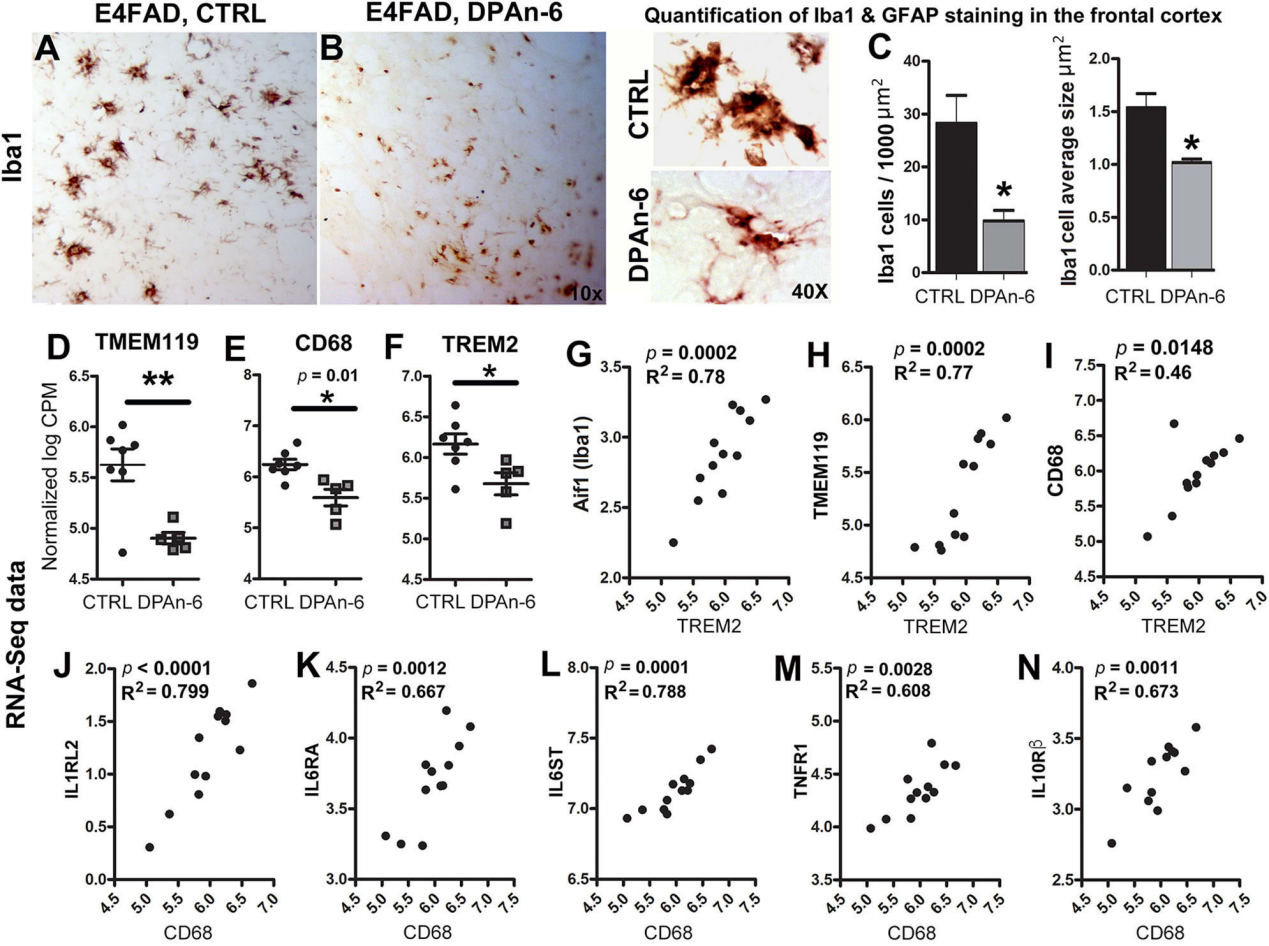
DPAn-6 attenuated microgliosis and reduced mRNA expression of microglial markers in aged E4FAD mice. **(A,B)** Immunostaining for the microglial marker Iba1 showed that DPAn-6 altered microglia morphology from an overactive hypertrophied shape to small ramified cell shape. **(C)** Quantification showed reduction of numbers of hypertrophied microglia and cellular size of activated microglia by DPAn-6 (*p* < 0.05). **(D–F)** DPAn-6 suppressed microglial gene expressions of TMEM119 (*p* < 0.01**, D**), CD68 (*p* = 0.01**, E**), and TREM2 (*p* < 0.05**, F**) compared to controls (CTRL) on standard diet. **(G–I)** TREM2 was positively correlated with Iba1 (Aif1, *p* = 0.0002, R^2^ = 0.78, **G**), TMEM119 (*p* = 0.0002, R^2^ = 0.77, **H**), and CD68 (*p* = 0.0148, R^2^ = 0.46, **I). (J–N)** Activated microglial marker CD68 was positively corelated with IL1RL2 (*p* < 0.0001, R^2^ = 0.799, **J**), IL6RA (*p* = 0.0012, R^2^ = 0.667, **K**), IL6ST (*p* = 0.0001, R^2^ = 0.788, **L**), TNFR1 (*p* = 0.0028, R^2^ = 0.608, **M**), IL10Rß (*p* = 0.0011, R^2^ = 0.673, **N**). The RNA-Seq data were normalized by Count Per Million (CPM) and presented as Log2 expression. Data represents the mean ± SEM. Significance was determined using an unpaired, two-tailed Student's *t*-test, *p < 0.05, and **p < 0.01.

Since microglia are primary CNS resident immune cells (46, 47) that consistently respond to Aβ deposits and/or neurodegeneration in AD to cause neuroinflammation and microgliosis (48), we further evaluated the impact of DPAn-6 on microglia. Figure 3 immunohistochemical staining of brain tissue sections with microglial marker Iba1 shows that DPAn-6 altered microglia morphology from overactive hypertrophied shapes toward small ramified morphology similar to functional “resting or surveillant microglia” (Figures 3A,B). DPAn-6 also quantitively reduced numbers of hypertrophied microglia and the size of activated microglial cells (*p* < 0.05, Figure 3C).

In support of the reduction of microgliosis, RNA-Seq data shows that DPAn-6 suppressed multiple innate immune specific microglial gene expression, including TMEM119 (*p* < 0.01, Figure 3D), CD68 (*p* = 0.01, Figure 3E) and TREM2 (*p* < 0.05, Figure 3F). Consistently, TREM2 was positively correlated with Iba1 (Aif1, *p* = 0.0002, R^2^ = 0.78, Figure 3G), TMEM119 (*p* = 0.0002, R^2^ = 0.77, Figure 3H) and CD68 (*p* = 0.0148, R^2^ = 0.46, Figure 3I). Iba1, expressed in both resting and activated microglia, had a trend toward reduction by DPAn-6 (*p* = 0.096, data not shown). Intriguingly, CD68, a specific marker for activated microglia in neuroinflammatory responses, was positively corelated with IL1RL2 (*p* < 0.0001, R^2^ = 0.799, Figure 3J), IL6RA (*p* = 0.0012, R^2^ = 0.667, Figure 3K), IL6ST (*p* = 0.0001, R^2^ = 0.788, Figure 3L), TNFR1 (*p* = 0.0028, R^2^ = 0.608, Figure 3M), and IL10Rβ (*p* = 0.0011, R^2^ = 0.673, Figure 3N). These data support coordinated gene upregulation in activated microglia and/or microgliosis with neuroinflammation in EFAD and their attenuation by DPAn-6 in aged E4FAD mice.

### Improvements of Neurodegeneration and Neuroprotection With DPAn-6 in Aged E4FAD Mice

**Figure 4:**
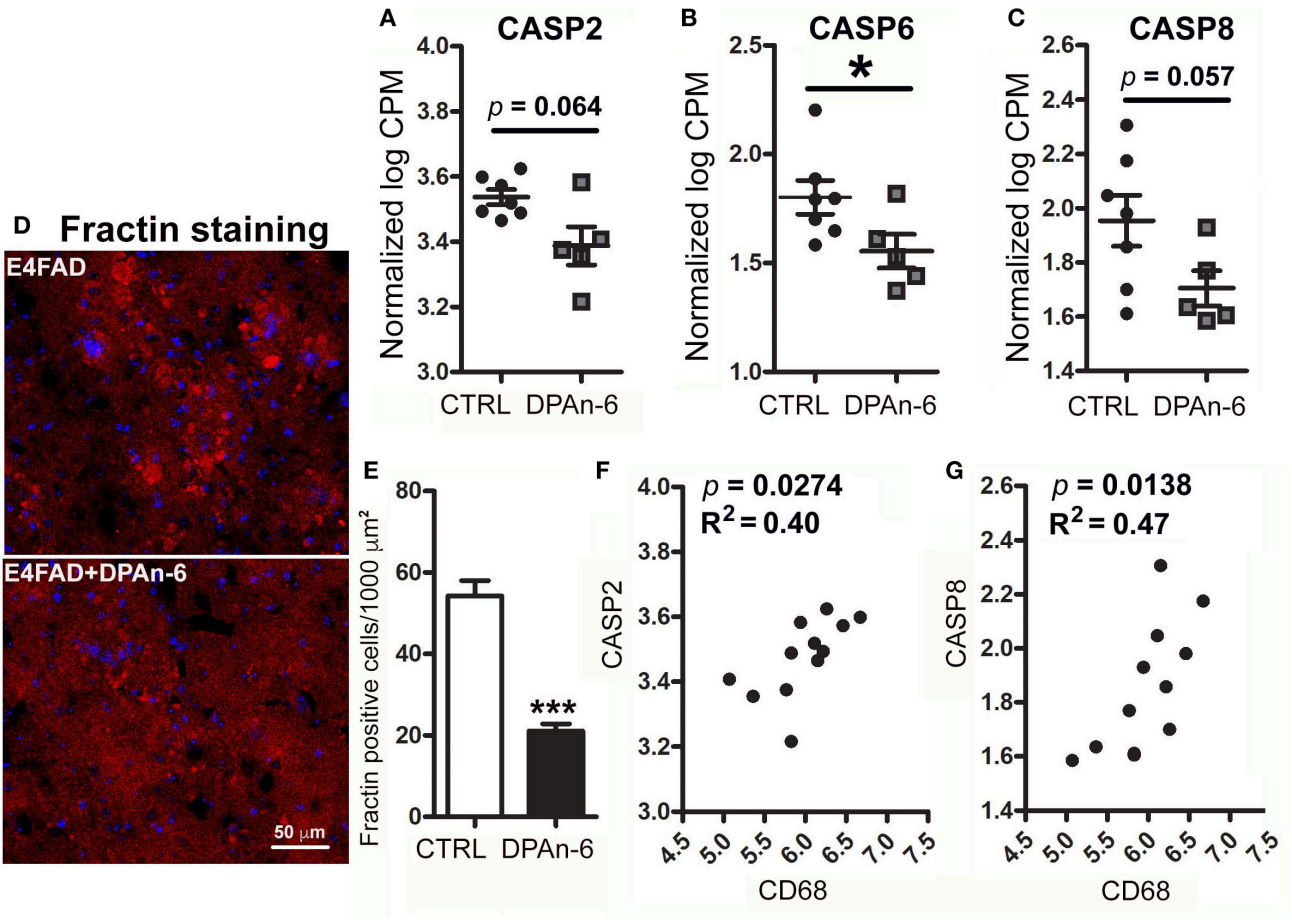
DPAn-6 reduced apoptosis in aged E4FAD mice. In RNA-Seq data, **(A–C)** DPAn-6 reduced gene expression of caspase 2 (CASP2, a trend *p* = 0.064, **(A)**), caspase 6 (CASP6, *p* < 0.05, **(B)**), and caspase 8 (CASP8, a trend *p* = 0.057, **(C)**) compared to controls (CTRL) on standard diet (ST). **(D,E)** Immunofluorescence staining revealed that DPAn-6 reduced caspase-cleaved fragment of actin (Fractin (red), **D**; *p* < 0.001, **E**). Nuclei were stained by DAPI (blue). **(F,G)** Activated microglial marker CD68 was positively correlated with CASP2 (*p* = 0.0274, R^2^ = 0.40, **(F)**) and CASP8 (*p* = 0.0138, R^2^ = 0.47, **(G)**). The RNA-Seq data were normalized by Counts Per Million (CPM) and presented as Log2 expression. Data represent the mean ± SEM. Significance was determined using an unpaired, two-tailed Student's *t*-test, *p < 0.05, and ***p < 0.001.

Since neurodegeneration can be mediated by neuroinflammation in AD (49), to understand whether DPAn-6 improves neurodegeneration and/or neuroprotective function from the reductions of neuroinflammation and microgliosis, we analyzed apoptotic, neurotrophic, and synaptic markers in RNA-Seq data. Caspase function has been well established in apoptosis, a major form of programmed cell death (50), and is implicated in neurodegeneration of AD (51). Figure 4 shows that DPAn-6 resulted in reduction of mRNA expression of apoptotic markers of caspase 2 (CASP2, a trend, *p* = 0.064, Figure 4A), caspase 6 (CASP6, *p* < 0.05, Figure 4B), and caspase 8 (CASP8, a trend, *p* = 0.057, Figure 4C) in aged E4FAD mice. Consistent with the reduction of caspases, caspase-cleaved action (Fractin) was also reduced by DPAn-6 quantified by immunofluorescent staining (*p* < 0.001, Figures 4D,E). In addition, activated microglial marker CD68 was positively correlated with CASP2 (*p* = 0.0274, R^2^ = 0.40, Figure 4F) and CASP8 (*p* = 0.0138, R^2^ = 0.47, Figure 4G), consistent with the view that neuroinflammation and microgliosis may play important roles in apoptosis.

**Figure 5:**
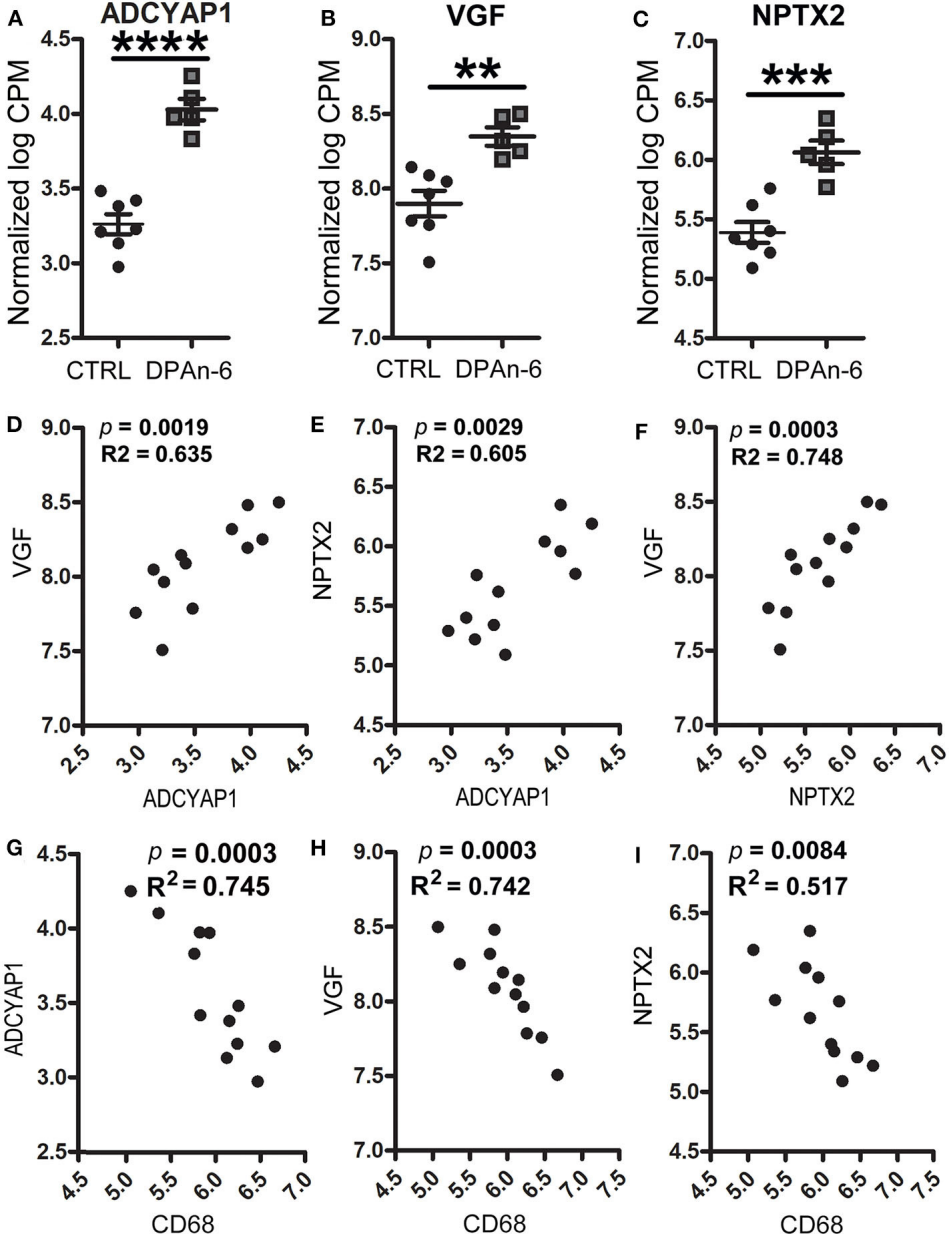
DPAn-6 increased mRNA expression of ADCYAP1, VGF, and NPTX2, which were inversely correlated with activated microglia in aged E4FAD mice. In RNA-Seq data, **(A-C)** DPAn-6 significantly increased gene expressions of ADCYAP1 (*p* < 0.0001, **A**), VGF (*p* < 0.01, **B**), and NPTX2 (*p* < 0.001, **C**). **(D–F)** These neuronal markers were positively correlated with each other. ADCYAP1 was positively correlated with VGF (*p* = 0.0019, R^2^ = 0.635, **D**) and NPTX2 (*p* = 0.0029, R^2^ = 0.605, **E**). NPTX2 was positively correlated with VGF (*p* = 0.0003, R^2^ = 0.748, **F**). **(G–I)** The microglial activation marker CD68 is was inversely correlated with ADCYAP1 (*p* = 0.0003, R^2^ = 0.745, **G**), VGF (*p* = 0.0003, R^2^ = 0.742, **H**), and NPTX2 (*p* = 0.0084, R^2^ = 0.517, **I**). The RNA-Seq data were normalized by Count Per Million (CPM) and presented as Log2 expression. Data represent the mean ± SEM. Significance was determined using an unpaired, two-tailed Student's *t*-test, **p < 0.01, ***p < 0.001, and ****p < 0.0001.

In addition, we found DPAn-6 increased gene expression of neurotrophins and synaptic markers. Our data show that DPAn-6 increased mRNA expression of adenylate cyclase activating polypeptide 1 (ADCYAP1, *p* < 0.0001, Figure 5A), VGF nerve growth factor (VGF, *p* < 0.01, Figure 5B) and excitatory synaptic marker neuronal pentraxin-2 (NPTX2, *p* < 0.001, Figure 5C). ADCYAP1 encodes pituitary adenylate cyclase-activating polypeptide 1, a hypophysiotropic hormone that functions as both neurotransmitter and neuromodulator in the brain. ADCYAP1 gene expression is reduced in multiple regions of the human AD brain (52). VGF is a neuroprotective neuropeptide that can be increased by BDNF and regulates synaptic plasticity, including NPTX2 (53). Multiple studies have demonstrated the downregulation of VGF in the AD brain, CSF, and plasma (54–62). NPTX2 (also called Narp, neuronal activity–regulated pentraxin) is selectively enriched at excitatory synapses and plays an essential role in excitatory synaptogenesis by clustering AMPA Receptors (63). NPTX2 is markedly reduced in the AD brain and CSF (64).

**Figure 6:**
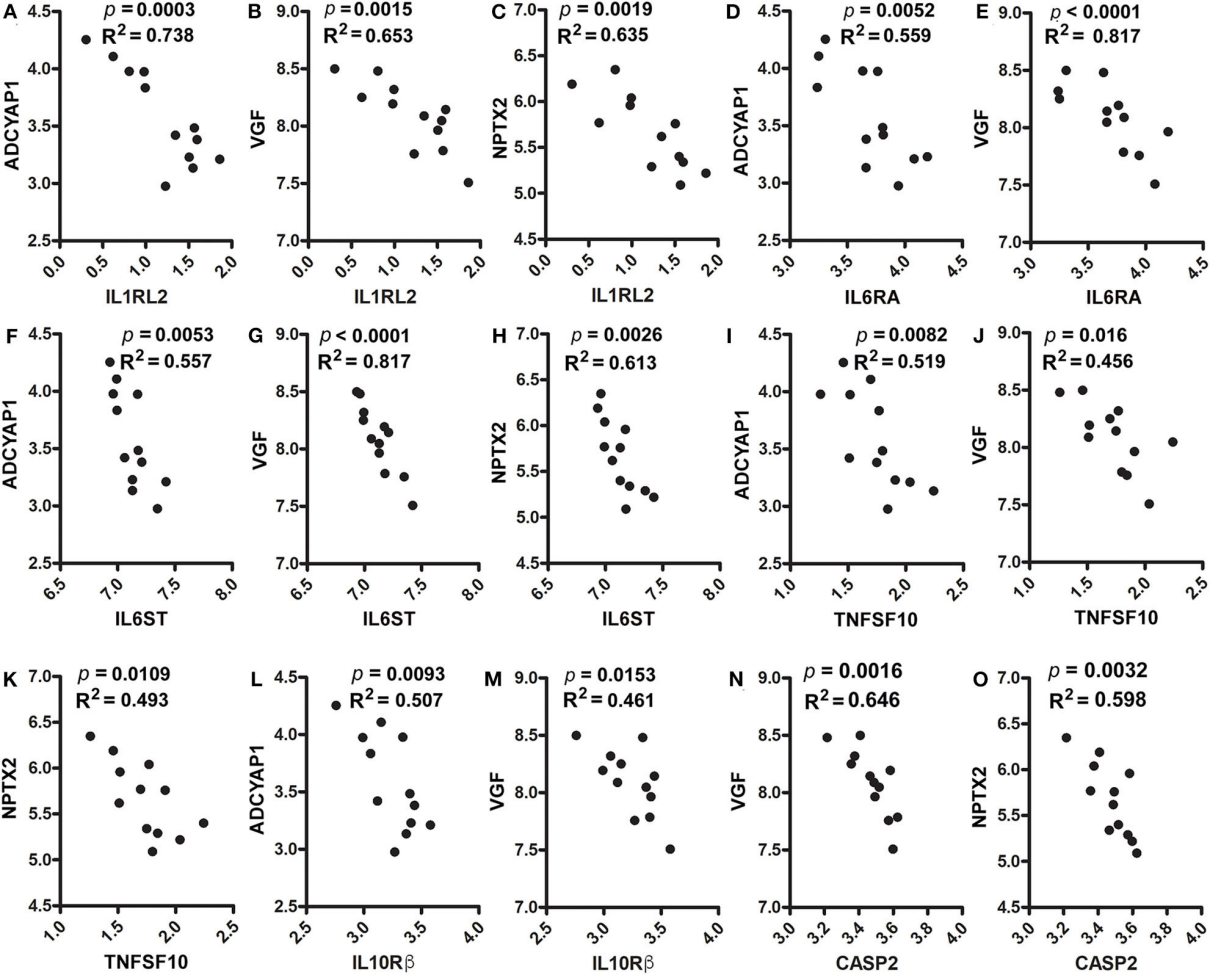
Correlation analysis of ADCYAP1, VGF, and NPTX2 with proinflammatory cytokines, cytokine receptors and caspases. In RNA-Seq data, **(A–C)**. IL1RL2 was inversely correlated with ADCYAP1 (*p* = 0.0003, R^2^ = 0.738, **A**), VGF (*p* = 0.0015, R^2^ = 0.653, **B**), and NPTX2 (*p* = 0.0019, R^2^ = 0.635, **C**). **(D,E)** IL6RA was inversely correlated with ADCYAP1 (*p* = 0.0052, R^2^ = 0.559, **D**) and VGF (*p* < 0.0001, R^2^ = 0.817, **E**). **(F–H)** IL6ST was inversely correlated with ADCYAP1 (*p* = 0.0053, R^2^ = 0.557, **F**), VGF (*p* < 0.0001, R^2^ = 0.817, **G**), and NPTX2 (*p* = 0.0026, R^2^ = 0.613, **H**). **(I–K)** TNFAF10 was inversely correlated with ADCYAP1 (*p* = 0.0082, R^2^ = 0.519, **I**), VGF (*p* = 0.016, R^2^ = 0.456, **J**), and NPTX2 (*p* = 0.0109, R^2^ = 0.493, **K**). **(L,M)** IL10Rß was inversely correlated with ADCYAP1 (*p* = 0.0093, R^2^ = 0.507, **L**) and VGF (*p* = 0.0153, R^2^ = 0.461, **M**). **(N,O)** CASP2 was inversely correlated with VGF (*p* = 0.0016, R^2^ = 0.646 **N**) and NPTX2 (*p* = 0.0032, R^2^ = 0.598, **O**).

Furthermore, these genes were positively corelated among each other. However, they were inversely correlated with cytokines, cytokine receptors, activated microglia and apoptosis markers. Figures 5D–F shows positive correlations between ADCYAP1 and VGF (*p* = 0.0019, R^2^ = 0.635, Figure 5D), and NPTX2 (*p* = 0.0029, R^2^ = 0.605, Figure 5E). NPTX2 was positively corelated with VGF (*p* = 0.0003, R^2^ = 0.748, Figure 5F). Figures 5G–I reveals inverse correlations between CD68 and ADCYAP1 (*p* = 0.0003, R^2^ = 0.745, Figure 5G), VGF (*p* = 0.0003, R^2^ = 0.742, Figure 5H), and NPTX2 (*p* = 0.0084, R^2^ = 0.517, Figure 5I). Figures 6A–C show inverse correlations between IL1RL2 and ADCYAP1 (*p* = 0.0003, R^2^ = 0.738, Figure 6A), VGF (*p* = 0.0015, R^2^ = 0.653, Figure 6B), NPTX2 (*p* = 0.0019, R^2^ = 0.635, Figure 6C). Figures 6D,E display inverse correlations between IL6RA and ADCYAP1 (*p* = 0.0052, R^2^ = 0.559, Figure 6D), VGF (*p* < 0.0001, R^2^ = 0.817, Figure 6E). Figures 6F–H show inverse correlations between IL6ST and ADCYAP1 (*p* = 0.0053, R^2^ = 0.557, Figure 6F), VGF (*p* < 0.0001, R^2^ = 0.817, Figure 6G), and NPTX2 (*p* = 0.0026, R^2^ = 0.613, Figure 6H). Figures 6I–K show inverse correlations between TNFSF10/TRAIL and ADCYAP1 (*p* = 0.0082, R^2^ = 0.519, Figure 6I), VGF (*p* = 0.016, R^2^ = 0.456, Figure 6J), NPTX2 (*p* = 0.0109, R^2^ = 0.493, Figure 6K). Figures 6L,M show the inverse correlations between IL10Rβ and ADCYAP1 (*p* = 0.0093, R^2^ = 0.507, Figure 6I), VGF (*p* = 0.0153, R^2^ = 0.461, Figure 6M). Figures 6N,O show the inverse correlations between CASP2 and VGF (*p* = 0.0016, R^2^ = 0.646 Figure 6N), NPTX2 (*p* = 0.0032, R^2^ = 0.598, Figure 6O). Taken together, these data suggest that the reduction of neuroinflammation and microgliosis with DPAn-6 is associated with improvements in indices of neurodegeneration and the protective VGF>NPTX2 pathway.

### Suppression of Cyclooxygenases (COXs) With Omega-6 Fatty Acids *in vivo* and *in vitro*

**Figure 7:**
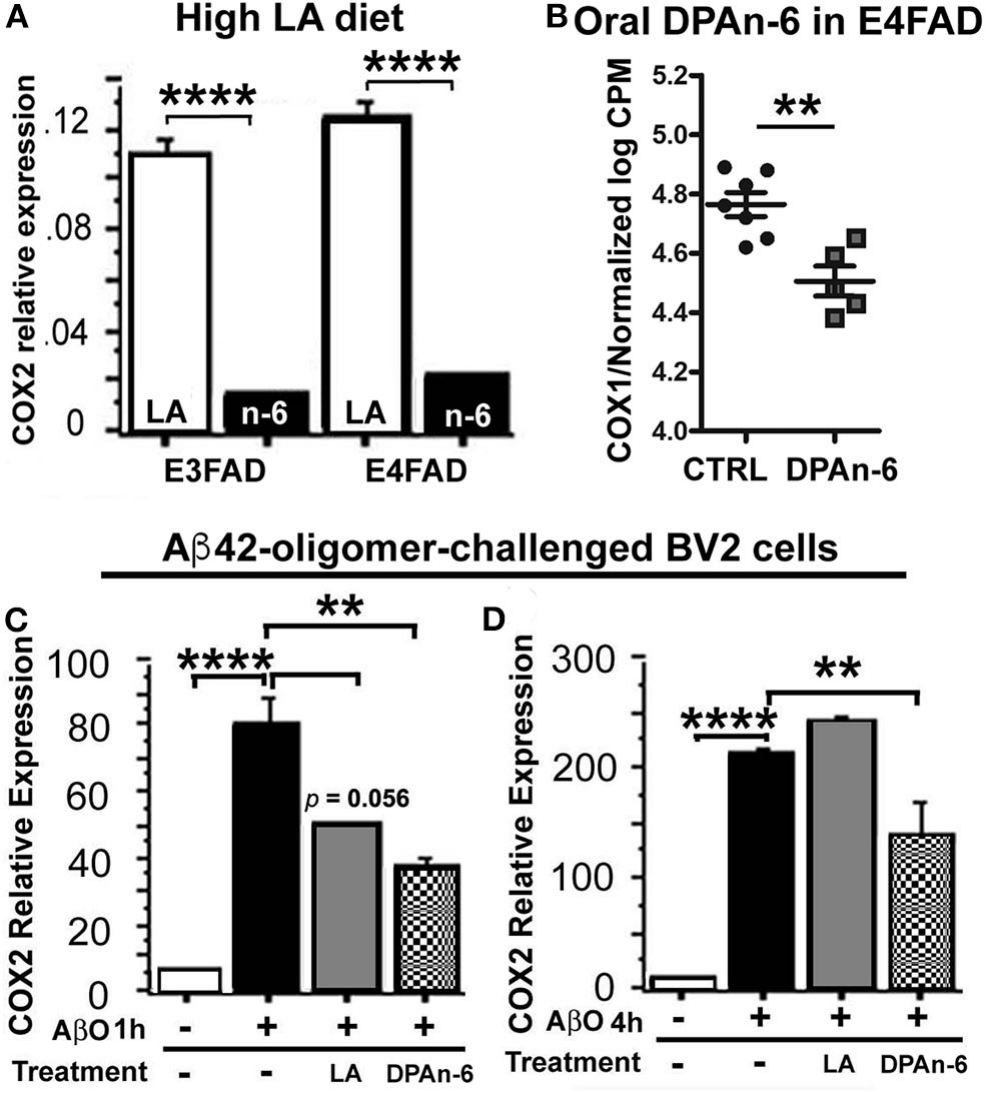
Linoleic acid and DPAn-6 reduced cyclooxygenases (COXs) in EFAD mice and in Aβ42 oligomers-stimulated microglial BV2 cells. **(A)** A high LA diet reduced mRNA expression of COX2 in both E3FAD and E4FAD mice compared to standard (ST) diet (*p* < 0.0001) measured by RT-qPCR. **(B)** DPAn-6 reduced COX1 gene expression in RNA-Seq data (*p* < 0.01) in DPAn-6 treatment of aged E4FAD. **(C,D)** DPA n-6 inhibited Aβ42 oligomer-stimulated elevation of COX2 mRNA measured by RT-qPCR at 1 h and 4 h compared to the Aβ42 oligomer-stimulated cells without DPAn-6 treatment (*p* < 0.01, **C,D**). LA showed a trend for short-term protective effects at 1 h (*p* = 0.056, **C**) but not at 4 h (*p* > 0.05, **D**). Data represent the mean ± SEM. Significance was analyzed by one-way ANOVA **(A,C,D)** and an unpaired, two-tailed Student's *t*-test **(B)**, **p < 0.01 and ****p < 0.0001.

A major negative concern with high n-6 linoleic acid (LA) intake has been the potential overproduction of eicosanoids derived from arachidonic acid metabolism via cyclooxygenases (COX) pathways, during which the inflammation is upregulated by eicosanoids. To test whether high LA diet influences COX, we measured brain COX2 mRNA expression by quantitative real-time RT-qPCR in EFAD mice fed with the high LA diet. Figure 7A shows that the high LA diet significantly suppressed COX2 mRNA expression in both E3FAD and E4FAD mice compared to standard (ST) diet (*p* < 0.0001). In the DPAn-6 experiment, DPAn-6 reduced COX1 gene expression in RNA-Seq data in aged E4FAD mice (*p* < 0.01, Figure 7B). COX1 elevation is more microglia-specific than COX-2 which is elevated in neurons in AD brains, so we measured the impact of DPA on COX-2 in a cultured microglial cell line. DPAn-6 inhibited Aβ42 oligomer-stimulated COX2 mRNA expression in cultured microglial BV2 cell lines *in vitro* measured by RT-qPCR. In this experiment, BV2 cells were pretreated with 50 μM of DPAn-6 or 50 μM of LA for 24 h followed by treatment with 0.25 μM of Aβ42 oligomers for an additional 1 or 4 h. We found that DPAn-6 inhibited Aβ42 oligomers-stimulated elevation of COX2 mRNA expression at both 1 and 4 h, compared to Aβ42 oligomer-challenged control group without DPAn-6 treatment (*p* < 0.01, Figures 7C,D). In contrast, LA only showed short-term protective effects at 1 h (*p* = 0.056, Figure 7C) but not at 4 h (*p* > 0.05, Figure 7D). These data indicate that high n-6 LA diet and DPAn-6 suppress, rather than increase, COX mRNA expression, thus providing one possible explanation for high n-6 fatty acids' prevention of neuroinflammation.

## Discussion

For decades, omega-6 (n-6) polyunsaturated fatty acids (PUFAs) have been widely considered pro-inflammatory. However, in this study, we provide initial evidence that a high dietary n-6 linoleic acid (LA, 18:2n-6) or oral gavage of its metabolite n-6 docosapentaenoic acid (DPAn-6, 22:5n-6) actually promote resolution of neuroinflammation, suppressing microglia activation and cyclooxygenases (COXs), and improving indices of apoptotic neurodegeneration and the neuroprotective VGF pathway implicated in neurons in Alzheimer's disease (AD) model 5xFAD/APOE mice, including E3FAD and E4FAD mice.

Extensive studies have suggested that n-6 PUFAs are primarily pro-inflammatory because n-6 arachidonic acid (ARA, 20:4n-6) is a substrate for COX enzymes that produce prostaglandins, well-known important inflammatory mediators. While LA is a precursor of ARA, unexpectedly, here both dietary LA and oral gavage DPAn-6 reduced COX gene expression in AD models *in vivo* and in Aβ stimulated BV2 microglia *in vitro* (Figure 7). Our results are consistent with a study using LPS-stimulated human peripheral blood mononuclear cells where DPAn-6 also reduced COX-2 and the production of prostaglandin E2 (45). In fact, several studies have indicated that there is insufficient evidence supporting the common belief that high LA intake is harmful due to causing inflammation. A review paper that summarized 15 randomized controlled trials of dietary n-6 PUFAs (mainly LA), reported that none of the studies showed elevation of pro-inflammatory markers by n-6 PUFAs in healthy adult populations (65). Further, there is no conclusive evidence available regarding a safe upper limitation of LA intake (66). In addition, our data are also supported by clinical studies, in which high LA intake was associated with anti-inflammatory effects in healthy adults and in chronic inflammatory-related diseases such as allergic airway inflammation and periodontitis (16, 67–69). The underlying mechanisms remain unclear. Here we found that a high LA diet did not alter brain LA levels. Instead, it increased its metabolite DPAn-6 in the brain of AD models (Table 1), indicating LA could function through the very large percentage changes in its long chain metabolite, DPAn-6.

Although neuroinflammation plays a vital role in the development and progression of AD (70), we lack an effective treatment to control neuroinflammation relevant to AD. In human studies, n-6 PUFAs could have robust anti-neuroinflammatory effects toward resolution at both early and late disease stages as they do in our mouse AD models. In human, the neuroinflammatory phenotype occurs more robustly at earlier rather than later stages of AD (71). Consistent with human studies, we found that unlike adult 6–8-month-old E4FAD mice, the aged E4FAD mice display extremely low expression levels of IL-6, TNF, and IL-10, which were not even detectable in our RNA-seq data. IL-1 (IL1α and IL1ß) were barely detectable (Value of IL1α mRNA expression counts was 0.128~0.353, IL1ß was−1.68~−1.75, data not shown). This indicates that the elevation of these cytokines which can be detected at both the protein and mRNA level in younger animals was more pronounced at early rather than late stages of E4FAD mice. In human AD, pro-inflammatory cytokine levels also decline as the disease progresses, apparently reflecting a “tolerance” to the amyloid burden. This might explain why the reduction of neuroinflammation by NSAIDs has been ineffective in treating AD in clinical trials but repeatedly associates with reduced AD risk in epidemiological studies. Intriguingly, unlike NSAIDs, we found that DPAn-6 modulated neuroinflammation toward resolution in the late stage of aged E4FAD mice. DPAn-6 reduced mRNA expressions of IL1RL2, IL6RA, IL6ST, TNFR2, TRAIL, and IL10Rß (Figure 2), which are all significantly upregulated in AD brain (36, 37, 40, 42).

Microgliosis is one of the important pathological features of AD. We found that DPAn-6 attenuated microgliosis revealed by immunostaining. It also reduced gene expression of microglial markers of TMEM119, CD68, and TREM2 (Figure 3). TREM2 was correlated with Iba1, TMEM119, and CD68, consistent with TREM2 upregulation in specific phenotypes of disease or amyloid-associated microglia. TREM2 is a key player in the sustained-microglial expansion during aging (72) although its function is not yet fully understood. The genetic variants of TREM2 have been reported to increase AD risk (2, 3). TREM2 has been suggested as a receptor required for disease-associated microglia (DAM) (6). In support of this concept, TREM2 deficiency eliminates TREM2+ inflammatory macrophages and ameliorates pathology at later stages in AD mouse models (73). In addition, our data showed that the microglial activation marker CD68 was positively correlated with inflammatory markers of IL1RL1, IL6RA, IL6ST, TNFR1, and IL10Rß (Figure 3). Although numerous studies have demonstrated that activated microglia respond to phagocytosis of Aβ (74), however, after a prolonged period of activation, these activated microglia may decrease or lose their Aβ phagocytic capacity (75, 76). This may correlate with an Aβ “tolerant” phenotype that can be reversed by effective antibody therapies and treatments that reduce Aβ (77). Therefore, the improvement of microgliosis by DPAn-6 could improve microglial phagocytic function relevant to AD.

Furthermore, we found that DPAn-6 reduced gene expression of caspase 6 and trended to reduce caspase 2 and caspase 8 (Figure 4). These caspases are endoproteases that provide critical links in cell regulatory networks controlling inflammation and cell death, implicated in neurodegenerative diseases. Interestingly, the activated microglia marker CD68 was positively correlated with CASP2 and CASP8, indicating a close association of activated microglia with apoptosis in EFAD mice. DPAn-6 also reduced caspase-cleaved actin fragments (Fractin), further supporting DPAn-6 limiting apoptosis. In addition, DPAn-6 increased ADCYAP1 and the neurotrophic factor VGF as well as a known downstream excitatory synaptic protein marker, neuronal pentraxin 2 (NPTX2). They were inversely correlated with activated microglial marker CD68 (Figure 5), proinflammatory cytokine, cytokine receptors and apoptotic genes (Figure 6), consistent with a reciprocal relationship between a neurodegenerative “activated” microglial phenotype and the loss of excitatory synapses that occurs early in MCI.

**Figure 8:**
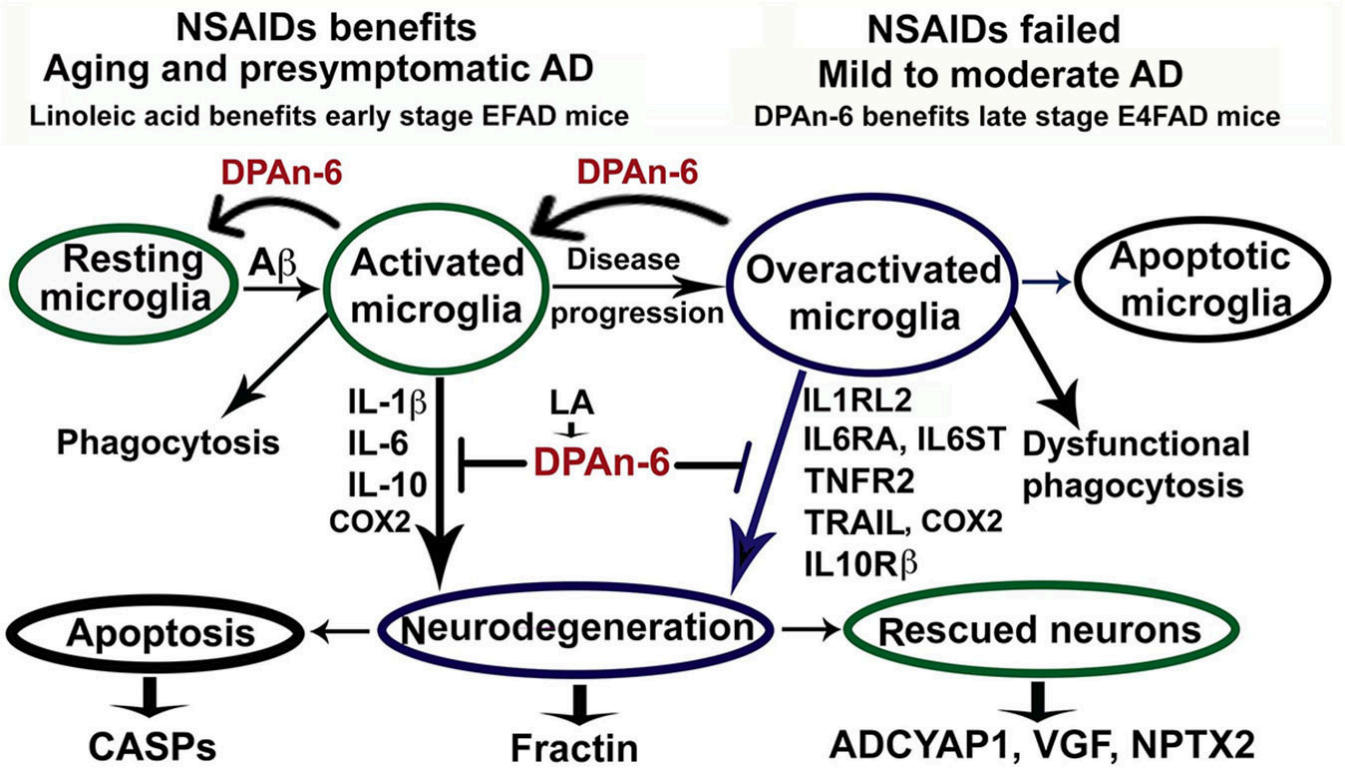
Schematic representation of omega-6 fatty acid docosapentaenoic acid (DPAn-6) positively resolving neuroinflammation in early and late stages of Alzheimer's disease (AD) models with the background of humanized APOE isoforms. In aging, MCI and early-stage AD, Aβ and other cellular debris can induce innate immune responses to activate microglia for phagocytosis (74). During this process, activated microglia release proinflammatory cytokines that can damage neurons if the response is not resolved adequately. Many studies have indicated that NSAIDs and other anti-inflammatory drugs, including COX2 inhibitors, can inhibit neuroinflammation in animal models (8, 9). However, clinical trials with these drugs have failed to date (12). Here we found that DPAn-6 resolved neuroinflammation in early-stage AD models, suggesting DPAn-6 may function similarly to NSAIDs. Further, with the progression of AD, sustainably activated microglia (overactivated microglia) may lose their phagocytotic function. These overactivated microglia may reduce expression of some known proinflammatory cytokines but sustain increases in cytokine receptors of IL1RL2, IL6RA, IL6ST, TNFR2, TRAIL, and IL10Rß (35–43), which may further damage neurons. Here we found that DPAn-6 suppressed the expression of these selected cytokines and cytokine receptors at late stages in the E4FAD model. It also improved toxicity evidenced by reductions of caspases and caspase-cleavage fragments. DPAn-6, which is normally produced in the liver and can be taken up by the brain in response to high n-6 diets, can resolve neuroinflammation at the late stage of AD models with advanced AD pathology in the background of APOE4 isoform, a strong genetic risk factor for AD.

In conclusion for this study, we found that n-6 fatty acid linoleic acid and its metabolite DPAn-6 can have robust anti-neuroinflammatory effects *in vivo* in AD models while linoleic acid may exert protective functions through increasing brain DPAn-6 levels. DPAn-6 by gavage reduced neuroinflammation, microgliosis, and apoptosis, and it improved neuroprotective gene expression in the late stage of our AD models with advanced AD pathology against the background of APOE4, a strong genetic risk factor for late-onset AD (Figure 8). These indicate that DPAn-6 could be a new potential anti-inflammatory lipid mediator for treating neuroinflammation in AD.

## Data Availability Statement

The RNA-Seq data was deposited to NCBI - GSE156936.

## Ethics Statement

The animal study was reviewed and approved by the Animal Research Committee, University of California, Los Angeles.

## Author Contributions

Q-LM contributed to the experimental design, funding, conducting experiments, analyzing data, preparing figures, and writing the first manuscript of the paper. GC and SF contributed to the experimental design and funding and paper writing. MM and MP contributed to the RNA-Seq assay. TS contributed to the RNA-Seq data processing and analysis. BT contributed to his expertise in RNA-Seq/RT-qPCR assistance. MJ contributed to the proinflammatory cytokine assay. CZ, PD, DC, and XG contributed to the conduction of animal experiments. FR and KC contributed to the immunohistochemistry. ZL contributed to funding in support CZ living in USA. All authors contributed to the article and approved the submitted version.

## Conflict of Interest

The authors declare that the research was conducted in the absence of any commercial or financial relationships that could be construed as a potential conflict of interest.
